# Highly diverse population of *Picornaviridae* and other members of the *Picornavirales*, in Cameroonian fruit bats

**DOI:** 10.1186/s12864-017-3632-7

**Published:** 2017-03-23

**Authors:** Claude Kwe Yinda, Roland Zell, Ward Deboutte, Mark Zeller, Nádia Conceição-Neto, Elisabeth Heylen, Piet Maes, Nick J. Knowles, Stephen Mbigha Ghogomu, Marc Van Ranst, Jelle Matthijnssens

**Affiliations:** 1 Department of Microbiology and Immunology, Rega Institute for Medical Research, Laboratory of Viral Metagenomics, KU Leuven - University of Leuven, Leuven, Belgium; 2 Department of Microbiology and Immunology, Rega Institute for Medical Research, Laboratory for Clinical and Epidemiological Virology, KU Leuven - University of Leuven, Leuven, Belgium; 3 Department of Virology and Antiviral Therapy, Jena University Hospital, Friedrich Schiller University, Jena, Germany; 4 The Pirbright Institute, Ash Road, Pirbright, Woking, Surrey GU24 0NF UK; 5 Department of Biochemistry and Molecular Biology, Biotechnology Unit, Molecular and cell biology laboratory, University of Buea, Buea, Cameroon

**Keywords:** Bat, *Picornavirales*, Picornaviruses, Virome, Unbiased sequencing

## Abstract

**Background:**

The order *Picornavirales* represents a diverse group of positive-stranded RNA viruses with small non-enveloped icosahedral virions. Recently, bats have been identified as an important reservoir of several highly pathogenic human viruses. Since many members of the *Picornaviridae* family cause a wide range of diseases in humans and animals, this study aimed to characterize members of the order *Picornavirales* in fruit bat populations located in the Southwest region of Cameroon. These bat populations are frequently in close contact with humans due to hunting, selling and eating practices, which provides ample opportunity for interspecies transmissions.

**Results:**

Fecal samples from 87 fruit bats (*Eidolon helvum* and *Epomophorus gambianus*), were combined into 25 pools and analyzed using viral metagenomics. In total, *Picornavirales* reads were found in 19 pools, and (near) complete genomes of 11 picorna-like viruses were obtained from 7 of these pools. The picorna-like viruses possessed varied genomic organizations (monocistronic or dicistronic), and arrangements of gene cassettes. Some of the viruses belonged to established families, including the *Picornaviridae*, whereas others clustered distantly from known viruses and most likely represent novel genera and families. Phylogenetic and nucleotide composition analyses suggested that mammals were the likely host species of bat sapelovirus, bat kunsagivirus and bat crohivirus, whereas the remaining viruses (named bat iflavirus, bat posalivirus, bat fisalivirus, bat cripavirus, bat felisavirus, bat dicibavirus and bat badiciviruses 1 and 2) were most likely diet-derived.

**Conclusion:**

The existence of a vast genetic variability of picorna-like viruses in fruit bats may increase the probability of spillover infections to humans especially when humans and bats have direct contact as the case in this study site. However, further screening for these viruses in humans will fully indicate their zoonotic potential.

**Electronic supplementary material:**

The online version of this article (doi:10.1186/s12864-017-3632-7) contains supplementary material, which is available to authorized users.

## Background

The order *Picornavirales* consists of viruses with a monopartite or bipartite positive strand RNA genome of the families *Dicistroviridae, Iflaviridae, Marnaviridae, Secoviridae, Picornaviridae*, the genera *Bacillarnavirus* and *Labyrnavirus* as well as many other proposed unassigned species, all of which possess small non-enveloped icosahedral virions of approximately 30 nm in diameter with a pseudo T = 3 symmetry [1, 2]. A major characteristic of this order includes the presence of a three-domain replication block (Hel-Pro-Pol domains including a superfamily III helicase, a chymotrypsin-like proteinase and an RNA-dependent RNA polymerase, respectively). The genomic organization of members of this order is variable. In the families *Picornaviridae* and *Iflaviridae*, the replication block is found in the C-terminal region of the polyprotein, while the structural proteins are in the N-terminal region, all encoded within a monocistronic genome (except for members of the dicistronic *Dicipivirus* genus of the family *Picornaviridae)*. In others, such as the *Marnaviridae* and *Dicistroviridae*, the reverse order is true and can either be on a single (monocistronic) open reading frame (*Marnaviridae*) or on two separate (dicistronic) open reading frames (ORFs) (*Dicistroviridae, Bacillarnavirus, Labyrnavirus)* [3]. The family *Secoviridae* contains viruses with either monopartite genomes with the replication block at the N-terminus or bipartite genomes [4]. Of all, only viruses of the family *Picornaviridae* have been implicated in human and other vertebrate diseases such as gastroenteritis, meningitis, encephalitis, paralysis (non-polio and polio-type), myocarditis, hepatitis, upper respiratory tract infections and diabetes [5, 6].

Recently, members of the order *Picornavirales* have been discovered in stools of different animals: **fe**line **s**tool-**a**ssociated RNA **virus** (fesavirus) from cats in an animal shelter [7]; **fi**sh **s**tool-**a**ssociated RNA **virus** (fisavirus) in the intestinal content of a carp [8]; **po**rcine **s**tool-**a**ssociated RNA **viruses** (posaviruses) in the feces of healthy pigs [9, 10] and **hu**man **s**tool-**a**ssociated RNA **viruses** (husavirus) in stools of HIV positive and negative patients [11]. Moreover, the RdRP of most of these novel viruses clustered with a cDNA sequence found in *Ascaris suum* [12], suggesting that this cDNA would have come from a virus infecting the parasite [9]. Viruses of this order are known to infect vertebrates, arthropods, higher plants, fungi and algae. Here we report the identification and genomic characterization of 11 highly diverse near/complete picorna-like genomes from *Eidolon helvum* fruit bat.

## Methods

### Sample collection and preparation

Fecal samples were collected from fruit bats between December 2013 and May 2014 as previously described in [13, 14]. Briefly, bats were captured in three different regions (Lysoka, Muyuka and Limbe) of the South West Region of Cameroon using mist nets around fruit trees. Each bat was held in paper sack for 5 to 20 minutes allowing enough time for fresh feces to be produced after which the bats were released. Then, 25 pools were made from 87 collected samples (85 from *Eidolon helvum* and 2 from *Epomophorus gambianus)* and the pools were treated to enrich for viral particles using the NetoVIR protocol [15]. Sequencing of the samples was performed on a HiSeq 2500 platform (Illumina) for 300 cycles (2x150 bp paired ends). Additional PCRs (list of primers can be found in Additional file 1: Table ﻿S1) and Sanger sequencing (Applied Biosystems) was performed to: 1) complete gaps in the obtained genomes, or 2) to verify genomic regions covered by less than 5 sequence reads.

### Genomic and phylogenetic analysis

NGS reads were analyzed as described in Yinda et al. [13, 14]. Briefly, after raw reads were trimmed, *de novo* assembled and contigs annotated, open reading frames (ORF) were identified and further analyzed for conserved motifs identification in the amino acid sequences using NCBI's conserved domain database (CDD) [16], Pfam [17] and Phyre2 [18]. Nucleotide alignments of the viral sequences were done with Muscle implemented in MEGA5 and adjusted manually [19]. For phylogenetic tree inference, four Bayesian Metropolis-coupled Markov chains were calculated with MrBayes version 3.1.2 [20, 21] using an optimal substitution model. Sequences used in the phylogenetic analysis were representatives of the different families or unassigned members of the order *Picornavirales*. All sequences were deposited in GenBank under the accession numbers KX644936-KX644946. Raw reads were submitted to the NCBI’s Short Read Archive (SRA) under the project ID PRJNA344863 (https://www.ncbi.nlm.nih.gov/bioproject/PRJNA344863/).

### Nucleotide composition analysis (NCA)

To infer the host of the viruses we identified, we used NCA as described in [22]. Briefly, all complete picorna-like genomes (segmented and non-segmented) whose host information is known were retrieved from Virus-Host DB [23] on the 1st of October 2016. Segmented genomes were concatenated together and treated as a single genome. Subsequently, all viruses that infect multiple hosts were removed, and only those infecting mammals, plants or insects were retained, resulting in a list of 351 genomes. A list of accession numbers for genomes used in NCA analysis is provided in Additional file 1: Table S2. Mononucleotide and dinucleotide frequencies for each genome were determined using an in-house developed python script. From these frequencies, the dinucleotide bias was calculated as described in [24]. Discriminant analysis was performed and the figure was created in R [25] using the MASS [26] and ggplot2 [27] packages, respectively. Briefly, the mononucleotide frequencies and dinucleotide bias for all possible dinucleotide combinations were used as discriminators. Furthermore, we used the predict function from the stats package [25]. The classification and posterior probabilities were calculated using the predicted linear discriminant analysis (LDA) model.

## Results

Illumina sequencing of 25 bat pools generated approximately 696 thousand non-phage viral reads of which 10% were assigned to the order *Picornavirales* (other viruses include those of the order/family *Astroviridae*, *Caliciviridae*, *Circoviridae, Coronaviridae*, *Papillomaviridae*, *Partitiviridae*, *Picobirnaviridae, Parvoviridae, Reoviridae, Tymovirales,* Table 1). Six of the pools (P6, P7, P12, P13, P19 and P20) had no reads for viruses of the *Picornavirales* order while pool 11 (P11) had the highest number of reads (52 thousand reads, 86.5% of the non-phage viral reads) (Table 1). We were able to obtain 11 (near) complete genomes of these viruses, which we named: bat kunsagivirus, bat crohivirus, bat sapelovirus, bat iflavirus (slow bee paralysis virus-like), bat posalivirus (posavirus-like), bat fisalivirus (fisavirus-like), bat cripavirus (Himetobi P virus-like), bat felisavirus (fesavirus-like), bat dicibavirus (JP-A-like), bat badiciviruses 1 and 2 (Aphis glycine virus-like) (explanation of the novel names is found in Table 2). Additionally, reads specific to kobuvirus (aichivirus A, mouse and Eidolon helvum kobuvirus), teschovirus, boone cardiovirus, torradovirus, sequivirus, waikavirus, and cripavirus/aphid lethal paralysis virus were detected. The number of reads of the (near) completely sequenced viral genomes ranged from 893 to 85,849. The number of reads for each of these viral genomes and the percentage of picorna-like reads from the total non-phage viral reads present in each pool can be found in column 3 and 4 of Table 1. All these sequences were substantially divergent from their closest known relatives and in the cases were closely related reference genomes were lacking, we are not certain of the completeness of the 5’-ends.

**Table 1:** Number of reads belonging to members of the order *Picornavirales* and percentages to total number of non-phage viral reads

Pool	Total non-phage viral reads	Picorna-like reads	% Picorna-like reads	Virus described	Viral reads percentage of picorna-like reads	Other viruses found in the pool (see figure legend)
P1	7068	178	2.5	-	-	2, 9 and 12
P2^a^	71955	2964	4.1	bat kunsagivirus	30.1	1, 9 and 11
				bat posalivirus	44.4	
				bat dicibavirus	25.5	
P3	82029	936	1.1	-	-	9 and 14
P4^a^	72953	8951	12.3	bat felisavirus	95.7	4, 6, 7, 9, 12 and 15
P5	1206	7	0.6	-	-	8, 9, and 16
P6	450	0	0	-	-	9
P7	7156	0	0	-	-	4, 9, 11, 14 and 15
P8	53007	613	1.2	-	-	11, 14and 15
P9	20866	70	0.3	-	-	2 and 4
P10	46295	35	0.1	-	-	2, 4, 6, 11and 15
P11^a^	60856	52661	86.5	bat badicivirus 1	98.2	4, 6 and 15
P12	787	0	0	-	-	2, 4, 6, 11 and 15
P13	2016	0	0	-	-	4, 6 and 15
P14	50567	1032	2	-	-	2, 3, 12 and 17
P15	108737	747	0.7	-	-	3, 12, 15 and 17
P16^b^	110542	586	0.5	-	-	3 and 9
P17^a^	227525	90976	40	bat iflavirus	94.4	9, 11 and 14
				bat cripavirus	3.7	
P18	1372	18	1.3	-	-	9, 11, 14, 16 and 17
P19	4334	0	0	-	-	9, 11, 12, 14 and 17
P20	626	0	0	-	-	4, 6, 11, 14, 15, and 17
P21	19222	1331	6.9	-	-	4, 6, 11, 14, 15 and 17
P22^a^	569516	6705	1.2	bat badicivirus 2	35.6	2 and 17
				bat fisalivirus	41.9	
P23	620965	1351	0.2	-	-	4
P24^a^	3107	1214	39.1	bat sapelovirus	83.7	1, 2, 3, 5, 11, 14, 15 and 17
P25^a^	15355	1319	8.6	bat crohivirus	100	3, 4, 8, 11, 14, 15 and 17

^a^Indicate pool from which (near) complete picorna-like virus genomes are described; − indicate cells corresponding to pools without picorna-like sequences or picorna-like viruses described. ^b^only pool with *Epomophorus gambianus* samples. Other viruses present are numbered as follows: 1 = *Astroviridae*, 2 = *Caliciviridae*, 3 = *Circoviridae*, 4 = *Coronaviridae*, 5 = *Hepeviridae*, 6 = *Herpesviridae*, 7 = *Nodaviridae*, 8 = *Papillomaviridae*, 9 = *Partitiviridae*, 10 = *Paramyxoviridae*, 11 = *Parvoviridae*, 12 = *Picobirnaviridae*, 14 = *Reoviridae*, 15 = *Retroviridae*, 16 = *Totiviridae*, 17 = *Tymovirales*

**Table 2:** Explanation of novel proposed names

Name of virus	Explanation
Posalivirus	**Posa**-**li**ke **virus**
Fisalivirus	**Fisa**-**li**ke **virus**
Felisa-virus	from fesa-like virus (Fesalivirus) to avoid confusion with fisalivirus
Badicivirus	**ba**t **dici**stro**virus**
Dicibavirus	anagram from bat dicistrovirus because this virus has exchanged CP and Hel-Prot-Pol domains compared to badicivirus

**Fig. 1:**
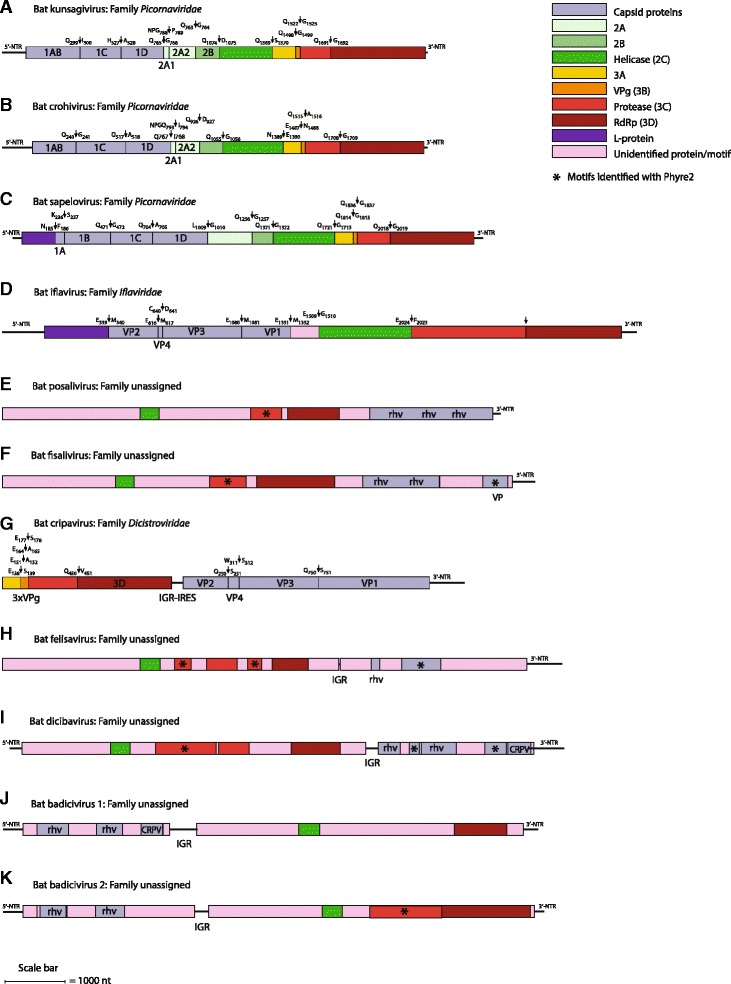
The genome organization and conserved picornaviral motifs of (**a**) bat kunsagivirus; (**b**) bat crohivirus; (**c**) bat sapelovirus; (**d**) bat iflavirus; (**e**) bat posalivirus; (**f**) bat fisalivirus 2; (**g**) bat cripavirus; (**h**) bat felisavirus; (**i**) bat dicibavirus; (**j**) bat badicivirus 1 and (**k**) bat badicivirus 2. The proposed cleavage sites of bat kunsagivirus, crohivirus, sapelovirus and iflavirus are indicated with arrows. Conserved motifs were identified with CDD [16], Pfam [17], and Phyre2 [18]. Proposed cleavage sites of the polyprotein of kunsagivirus, crohivirus, sapelovirus and iflavirus are indicated with arrows and amino acid position

The organization of these viral genomes varied greatly. Bat kunsagivirus, bat crohivirus, bat sapelovirus, bat iflavirus, bat posalivirus and fisalivirus possess monocistronic genomes, whereas bat cripavirus, bat felisavirus, bat dicibavirus and bat badiciviruses 1 and 2 all possess dicistronic genomes (Fig. 1).

**Fig. 2:**
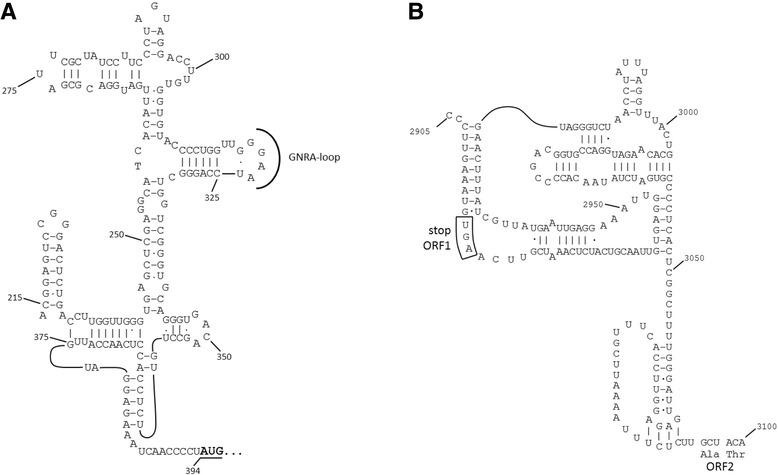
Proposed type IV (HCV-like) IRES of bat kunsagivirus (**a**) and IGR IRES of bat cripavirus (**b**)

The bat kunsagivirus genome exhibits a genome organization typical for members of the family *Picornaviridae* and it is at least 7092-nt long with a single ORF (7050 nt) flanked by at least a 393-nt long 5’ UTR and a 41-nt long 3’ UTR (Table 3 and Fig. 1a). Although it is likely that approximately 100 nucleotides at the 5’-end of the bat kunsagivirus genome are still missing, the similarity of the type IV IRES structures of roller kunsagivirus proposed in [21] and the bat kunsagivirus (Fig. 2a) is evident. The ORF displays the typical picornaviral genome layout with the Hel-Pro-Pol domains located downstream from the picornaviral structural protein domains (Fig. 1a). The proposed proteolytic cleavage sites of bat kunsagivirus were mapped based on the alignment with the monocistronic European roller kunsagivirus (roller/SZAL6-KuV/2011/HUN) sequence (KC935379) and they are shown in Fig. 1a (with arrows). The deduced polyprotein precursor has three capsid proteins (1AB, 1C, 1D ranging from aa 1 to 299, 300 to 527 and 528 to 765, respectively) each with a conserved picornavirus-like capsid protein (rhv) domain. The polyprotein lacks both an obvious leader polypeptide and a myristoylation signal found in many picornaviruses. Similar to the arrangement found in the genome of roller kunsagivirus (KC935379), two gene regions encoding 2A proteins were predicted: 2A1 is a 23 aa long aphthovirus-like protein whereas 2A2 is 175 aa long without similarity to known proteins. The bat kunsagivirus 2A2 was 10 amino acids longer than the roller kunsagivirus 2A2 (175 vs. 165 aa) and did not exhibit the conserved amino acids of the protein kinase C superfamily as suggested previously [28]. Likewise, no similarity of the 3 C-terminal sequence and 3B sequence was observed in the bat kunsagivirus questioning the significance of the previous findings which suggested existence of a conserved motif in this region [28]. The genome contained all of the conserved amino acid motifs of picornaviral 2C helicase, 3C proteinase and 3D RNA polymerase and showed the highest sequence identity (57% aa identity) to roller kunsagivirus in the 3D region (Table 4). However, the predicted bat kunsagivirus 3C is 19 aa shorter and 3D is 33 aa longer than the respective proteins of roller kunsagivirus. These combined findings suggest that the kunsagiviruses of bat and roller belong to different species.

**Table 3:** Genome characteristics of novel viruses and the corresponding representative species

Strain	Genome type	Genome size (#nt)	5’-UTR (#nt)	3’-UTR (#nt)	ORF (#aa)	IGR (#nt)
Bat kunsagivirus	monocistronic	7092	393	42	2218	NA
Kunsagivirus A1 (KC935379)	monocistronic	7272	500	25	2248	NA
Bat crohivirus	monocistronic	7081	508	54	2173	NA
Crohivirus (AB937989)	monocistronic	7321	517	293	2343	NA
Bat sapelovirus	monocistronic	7572	150	152	2481	NA
Sapelovirus (AF406813)	monocistronic	7491	443	51	2321	NA
Bat iflavirus	monocistronic	10520	28	273	3179	NA
Slow bee paralysis virus (NC_014137)	monocistronic	9505	316	293	2965	NA
Bat Posalivirus^a^	monocistronic	8343	-	128	2737	NA
Posavirus 1 (JF713720)	monocistronic	9840	829	152	2952	NA
Bat fisalivirus^a^	monocistronic	8862	-	382	2826	NA
Fisavirus (KM434233)	monocistronic	8712	395	57	2753	NA
Bat cripavirus^a^	monocistronic	6939	-	587	ORF1: >972 ; ORF2: 1233	190
Himetobi P virus (AB017037)	dicistronic	9275	963	588	ORF1: 1778 ; ORF2: 874	174
Bat felisavirus virus^a^	dicistronic	9299	-	588	ORF1: >1879 ; ORF2: 1006	23
Fesavirus 1 (KM017736)	monocistronic	6218	-	829	1743	NA
Bat dicibavirus	dicistronic	8882	120	249	ORF1: 1770; ORF2: 860	199
Marine JP-A virus (EF198241)	dicistronic	9236	632	434	ORF1: 1689; ORF2: 984	146
Bat badicivirus 1	dicistronic	8325	332	230	ORF1: 806; ORF2: 1623	459
Aphis glycines virus 1 (KF360262)	dicistronic	8305	-	113	ORF1: 776; ORF2: 1794	474
Bat badicivirus 2	dicistronic	8637	352	155	ORF1: 827; ORF2: 1806	229

- 5'UTR could not be obtained for this virus; #nt: number of nucleotides; #aa: number of amino acid; ^a^incomplete genomes

**Table 4:**
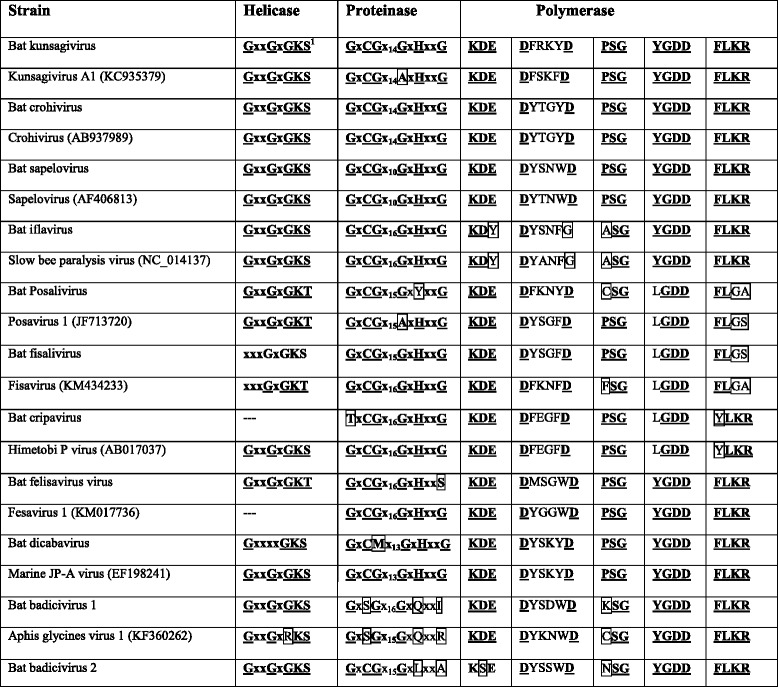
Genome functional motifs in the 3CD region of novel viral genomes and the corresponding representative species

Bat crohivirus (BtCroV) is the second monocistronic picornavirus identified and its genome showed the same organization as the yet unclassified shrew crohivirus and the genus *Pasivirus* (Fig. 1b) in the family *Picornaviridae*. We obtained a near complete genome of 7081 nt (Table 3), although it seems that a few nucleotides at the 5’ and 3’ ends are missing. The genome contains a predicted single large ORF encoding a putative polyprotein of 2173 aa, although the initiation codon (AUG) was not located in the Kozak consensus sequence (AAGAUGG) like in the CroV-1 genome [29]. The ORF is bordered by a 508 nt long 5’ UTR and 54 nt long 3’ UTR and the potential polyprotein cleavage sites are as shown in Fig. 1b. The BtCroV genome contained motifs of an aphthovirus-like 2A1 and a second 2A protein with unknown function. All of the conserved amino acid motifs of the picornaviral 2C helicase, 3C proteinase and 3D RNA polymerase were present (Table 4). The 3D polymerase showed 50% sequence identity to the shrew crohivirus (AB937989).

The third picorna-like sequence identified was a sapelovirus, exhibiting a genome length of at least 7572 nt with a single ORF of 2173 aa between at least a 20 nt 5’-UTR and 111 nt 3’-UTR ends (Table 3 and Fig. 1c). Based on prediction tools, the genome organization of bat sapelovirus showed similar features to sapelovirus A1 (former porcine sapelovirus 1) [30]. The predicted polyprotein is preceded by a leader protein of 185 aa, and has a long 2A protein of about 246 aa (20 aa longer than of sapelovirus A1). Most of the replication protein motifs are conserved (GxxGxGKS motif for nucleoside triphosphate binding sites of 2C; GxCGx_10_GxHxxG motif of 3C proteinase and the KDE, PSG, YGDD and FLKR motifs of RNA-dependent RNA polymerase) except DY**S**NWD of the RdRP that differs from sapelovirus A1 RdRp at position 3 (DY**T**NWD) (Table 4). Proposed proteolytic cleavage sites of bat sapelovirus were mapped based on the alignment with sapelovirus A1 (AF406813) and are shown in Fig. 1c with arrows.

We also identified sequences related to the slow bee paralysis virus of the family *Iflaviridae* tentatively named bat iflavirus. The genome is at least 10520 nt long and contains a 3179 aa ORF flanked by at least 28 nt and 273 nt at the 5’-UTR and 3’-UTR, respectively (Table 3). Like other iflaviruses, genes coding for structural proteins are located in the N-terminus of the ORF whereas those encoding the replication proteins are located at the C-terminal part (Fig. 1d). This genome organization is typical of members of the insect-specific virus family *Iflaviridae*, and more in particular for slow bee paralysis virus (SBPV, EU035616). Arrows in Fig. 1d indicate where the predicted polyprotein of bat iflavirus is processed into functional units based on an alignment with SBPV. The polyprotein is preceded by a leader protein of 176 aa at the N-terminus, followed by four capsid proteins. An unidentified protein of 148 aa (aa position 1155–1303) bridges the structural proteins and the replication block made up of Hel-Prot-Pol with the conserved motifs GxxGxGKS, GxCGx_16_GxHxxG, KDY, DYSNFG, ASG, YGDD and FLKR, respectively (Table 4).

Bat posalivirus and fisalivirus possess a single large ORF with the replication proteins at the N-terminus and capsid proteins at C-terminus (Fig. 1e and f). The 5’-ends of both viral sequences (5’-UTR and the N-terminal part of the polyprotein) are lacking. Posalivirus exhibits a genome length of at least 8341 nt with a long ORF of at least 8213 nucleotide (nt); the 3’-UTR is 127 nt long (Table 3). The genome layout corresponds to that of posaviruses 1 and 2 (JF713720, JF713721), fisavirus 1 (KM434233) and husavirus (KT215901, KT215902, KT215903). The posalivirus genome showed conserved domains of the RNA helicase (aa 766–870) and the RdRP (aa 1583–1867) (Fig. 1e). The NCBI BLASTP failed to detect conserved sequences that were unequivocally identified as protease domain. However, there is an amino acid stretch with the sequence GDCGX_15_GMY which is thought to extend the conserved GXCGX_n_GXH sequence motif of the peptidase C3 superfamily. Thus, it is likely that the deduced protein exhibits proteinase activity. There are three rhv-like domains, with domains 1 and 3 lacking conserved amino acids of the so-called “drug-binding pocket”. In addition, the genome contained motifs of multidrug and toxic compound extrusion (MATE) domains usually found only in bacteria [31]. The fisalivirus is similar to fisavirus 1 (KM434233) with a genome length of at least 8862 nt and a 382 nt non-coding region at the 3’-UTR. There is an ORF of at least 2420 aa with a genome organization like that of posalivirus except that it lacked sequences with detectable homology to a third rhv domain.

Apart from the monocistronic picorna-like viruses above, we also identified five *Picornavirales* genomes with a dicistronic organization.

Firstly, the bat cripavirus genome contained two predicted ORFs of 2919 nts (ORF1) and 3702 nts (ORF2) encoding replication proteins and structural proteins, respectively (Fig. 1g). There seemed to be a portion missing at the 5’ end of the ORF1. However, three copies of the genome-linked protein (VPg) at the aa positions 139–151, 152–164 and 165–177 as well as the consensus motifs for an RNA-dependent RNA polymerase (KDE, DFEGFD, PSG, LGDD and YLKR), were found in the deduced amino acid sequence (Table 4). The two ORFs are separated by a short intergenic region (IGR) of 190 nt that is supposed to form an internal ribosome entry site (IRES) (Fig. 2b) typical of the *Cripavirus* genus [32] and therefore clearly defines this virus as a cripavirus. ORF2 contains four predicted capsid protein motifs (three rhinovirus-like capsid protein domains (rhv) and one cricket paralysis virus (CRPV) capsid protein-like motif) at aa positions 1–250, 251–311, 312–750 and 751–1233, respectively.

The bat felisavirus is a dicistronic virus with a predicted ORF1 (5638 nt) encoding functional proteins and ORF2 (3020 nt) encoding capsid proteins. Both ORFs are separated by a very short intergenic region (IGR) of 73 nt (Fig. 1h). The 5’-end of the genome (5’ UTR and N-terminal sequences of the polyprotein) could not be obtained. The 3’ UTR has the remarkable length of 588 nt (Table 3). ORF1 exhibits conserved sequences of a P-loop NTPase (RNA helicase, 764–875 aa) with a G_768_XXGXGKT consensus sequence (Walker motif A), a proteinase (1129–1317 aa) with a conserved GXCGX_16_GXH motif and an RdRP (1510–1710 aa) with conserved KDE, PSG, YGDD and FLKR motifs (Table 4). ORF2 showed only a short stretch of 45 aa with weak similarity to a rhv domain (137–182 aa) (Fig. 1h).

Bat dicibavirus is a dicistronic virus that resembles viruses of the genus *Bacillarnavirus* which presumably infect diatoms. The ORF1 encodes functional proteins, containing conserved domains of an RNA helicase (aa 432–541) with a GX_4_GKS Walker A motif and a RsdvnGiyidD Walker B motif of P-loop NTPases, a proteinase (aa 1030–1198) with a GLCMX_13_GXH motif also found in other bacillarnaviruses and an RdRP (aa 1430–1701) with KDE, PSG, YGDD and FLKR motifs (Table 4). The ORF2 encodes two domains with homology to rhv (61–232 and 349–542 aa) and one with similarity to the CRPV (cricket paralysis virus) domain (827–955 aa) also found in dicistroviruses of insects. Rhv domain 1 also has some similarity to the calicivirus coat protein superfamily. Both ORFs are separated by a short intergenic region (IGR) of 196 nt (Fig. 1i). There seemed to be no RNA structures representing an IGR-IRES, however, ORF2 starts with a glycine codon which is typical of IGR-IRES driven translation initiation [33].

Surprisingly, two dicistronic picorna-like viruses with a 5’ ORF encoding capsid proteins and a 3’ ORF coding for the replication proteins were identified and provisionally named bat badiciviruses 1 and 2 (Fig. 1j, k). The bat badicivirus 1 ORF1 encodes three putative capsid proteins and is flanked by a 5’-UTR of at least 332 nt and an intergenic region of 459 nucleotides (Table 3) and ORF2 is followed to a 3’ UTR of 242 nucleotides (Fig. 1j). The capsid proteins exhibit similarity to rhv domains (aa 78–250 and aa 414–552) and the CRPV capsid superfamily (aa 602–687). In ORF2, only an RNA helicase domain (378–493 aa) and an RdRp domain (1241–1532) were predicted. Instead of a GXCGX_n_GXH motif of 3C-like proteinases, we observed the sequence GQSGX_16_GVAI suggesting an active site serine residue. The genome of bat badicivirus 2 comprises at least 8637 nt. The two ORFs are separated by a 229 nt IGR. ORF1 (333–2753 nt) encodes two conserved motifs of rvh (between 95–250 aa and 396–555 aa, respectively) while ORF2 (3061–8481 nt) was predicted to encode replication proteins: RNA helicase (634–743 aa), peptidase (2012–2290 aa) and an RdRP (1295–1785 aa). The 5’-UTR and 3’-UTR ends contain 352 and 155 nt, respectively.

**Fig. 3:**
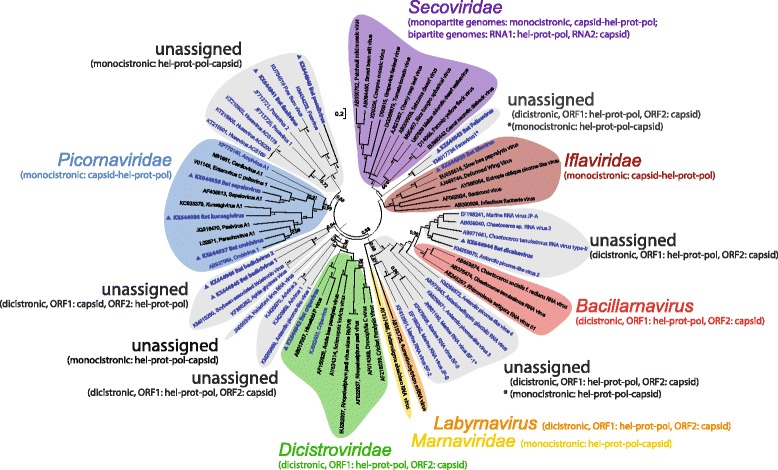
Phylogenetic relationships of bat kunsagivirus, bat crohivirus, bat sapelovirus, bat iflavirus, bat posalivirus, bat fisalivirus, bat cripavirus, bat felisavirus, bat dicibavirus, bat badicivirus and bat dicistronic virus (indicated with filled triangles), and representative and unassigned members of *Picornavirales* based on nucleotide sequences of the proteinase-polymerase domains. Bar indicates nucleotide substitutions per site. The tree was inferred with MrBayes 3.2. Numbers at nodes indicate posterior probabilities obtained after 3,000,000 generations using the GTR + G + I substitution model. Unassigned viruses are shown in blue

Phylogenetic analysis was performed using the nucleotide sequences of the proteinase-polymerase domains (3CD) of the eleven novel viruses, and 60 representative members of the order *Picornavirales* (Fig. 3). The phylogenetic tree confirmed the BLAST results and showed (i) clustering of bat kunsagivirus, bat crohivirus and bat sapelovirus with their cognate picornavirus genera [i.e., Kunsagivirus A1 (roller/SZAL6-KuV/2011/HUN, KC935379) Crohivirus 1 (shrew/ZM54/Zambia/2012, AB937989) and sapelovirus A1 (PEV8-V13, AF406813), respectively]; (ii) close clustering of bat iflavirus with slow bee paralysis virus (EU035616) and other members of the *Iflaviridae* (deformed wing virus, AJ489744; infectious flacherie virus, AB000906; sacbrood virus, AF092924; ectropis obliqua virus, AY365064); (iii) bat posalivirus and fisalivirus forming a clade of unassigned viruses comprising posaviruses 1 and 2 (JF713720 and JF713721), fisavirus 1 (KM434233), husavirus (KT215901-KT215903) and Pow Burn virus (KU754519); (iv) bat cripavirus, the only virus of the family *Dicistroviridae* described in this study formed a clade with cripavirus (NB-1/2011/HUN, KJ802403) and Himetobi P virus (AB017037) within the genus *Cripavirus,* family *Dicistroviridae;* (v) bat felisavirus, bat dicibavirus and bat badiciviruses 1 and 2 clustered to yet unassigned viruses. Bat felisavirus was closest to fesavirus 1 (KM017736) while bat badiciviruses 1 and 2 formed a cluster with Aphis glycines virus 1 (KF360262) and soybean-associated bicistronic virus (KM015260), with very low sequence identity even in the most conserved parts of the genome; (vi) finally, bat dicibavirus clustered with the diatom-specific Chaetoceros tenuissimus RNA virus type-II SS10-16 V (AB971661), Chaetoceros sp. RNA virus 2 Csp02RNAV01 (AB639040), marine RNA virus JP-A (EF198241), Asterionellopsis glacialis RNA virus (AB973945) and antarctic picorna-like virus 2 (KM259870), though clearly distant from these viruses (34-53% amino acid identity of 3D^pol^).

We further analyzed the phylogenetic relationships of the capsid proteins (Additional file 1: Figure S1). In order to ensure that only orthologous proteins were included, five distinct alignments including more relevant reference strains were investigated: (i) picornaviruses and candidates, (ii) bacillarnaviruses, labyrnavirus, bat dicibavirus and other dicistronic viruses with ORF2-encoded capsid proteins, (iii) insect dicistroviruses of the genera *Cripavirus* and *Aparavirus* and bat cripavirus, (iv) monocistronic viruses with C-terminal capsid proteins (husa-, posa-, fisaviruses, Pow Burn virus of *Drosophila subobscura*, bat fisalivirus and bat posalivirus), and (v) insect iflaviruses and bat iflavirus. Due to the lack of representative sequence data for comparison, bat badiciviruses 1 and 2 and the bat felisavirus are not shown.

### Nucleotide composition analysis (NCA)

**Fig. 4:**
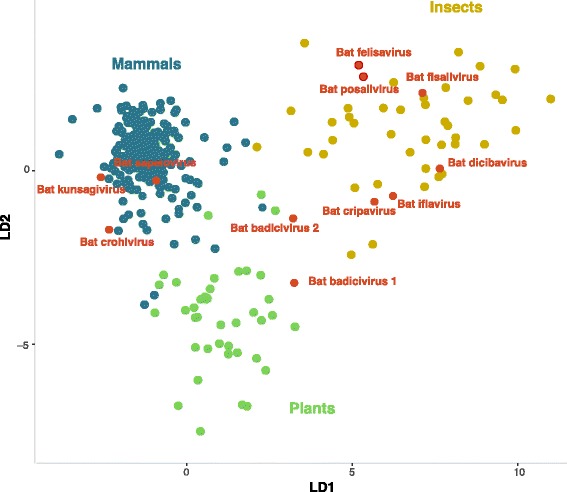
Linear discriminant analysis employed in the classification of viral sequences into host groups. Graphs represent separation of groups using the most influential factors and points represent values for individual sequences

Based on the mononucleotide and dinucleotide frequencies of a training dataset of 351 picorna-like viruses and their known host (mammalian, insects or plants) we performed a linear discriminant analysis (LDA) in an attempt to identify the most likely host of the viruses described in this paper. Based on the predicted LDA model, the classification and posterior probabilities of the viruses described in this paper were inferred (Fig. 4, Table 5). Bat kunsagivirus, sapelovirus and crohivirus clustered with picorna-like viruses that infect mammals, while bat badiciviruses 1 and 2 were found in the plant cluster. The rest of the viruses (bat felisavirus, fisalivirus, posalivirus, dicibavirus, iflavirus and cripavirus) clustered with picorna-like viruses known to infect insects. Posterior probabilities for the classification was greater than 0.9 for all newly identified viruses (Table 5).

**Table 5:** Posterior probability for the classification of the novel picorna-like sequences, based on linear discriminant analysis (LDA)

Virus	Mammals	Plants	Insects
Bat_Kunsagivirus	1.000	0	0
Bat_crohivirus	1.000	0	0
Bat_sapelovirus	1.000	0	0
Bat_iflavirus	0	0	1.000
Bat_posalivirus	0	0	1.000
Bat_fisalivirus	0	0	1.000
Bat_cripavirus	0	0	1.000
Bat_felisavirus	0	0	1.000
Bat_dicibavirus	0	0	1.000
Bat_badicivirus_1	0	1.000	0
Bat_badicivirus_2	0.043	0.914	0.044

## Discussion

Using the NetoVIR protocol for viral metagenomics, we could retrieve the (nearly) complete genomes of eleven diverse and highly divergent viruses belonging to the order *Picornavirales*. Six out of these eleven viruses are likely to represent members of yet unassigned virus families of this order whereas bat crohivirus, bat sapelovirus, bat kunsagivirus, bat iflavirus, and bat cripavirus, belong to the established families *Picornaviridae*, *Iflaviridae* and *Dicistroviridae*.

Picornaviruses of the family *Picornaviridae* identified here were bat kunsagivirus, bat crohivirus and bat sapelovirus. The genome layout of bat kunsagivirus exhibits close similarity to that of roller kunsagivirus (species *Kunsagivirus A*) of the family *Picornaviridae*, but its deduced amino acid sequence exhibits significant differences suggesting classification into a novel species (proposed name “Kunsagivirus B”). The host of roller kunsagivirus was unclear as several rodent-related viruses were also detected in the same fecal sample, e.g. mosavirus, murine kobuvirus and Puumala virus (21). Therefore, a dietary origin of roller kunsagivirus could not be excluded. In line with this dietary hypothesis, bat kunsagivirus is predicted to have a mammalian host by NCA. Therefore, we speculate that fruit eating bats are most likely the host of the kunsagivirus identified in this study. Similarly, crohivirus (CroV1, AB937989), first isolated from a Zambian shrew [29] is a yet unclassified picornavirus with a pasivirus-like genome organization. The bat crohivirus has the same genome organization and shares the same features with the one from a shrew, although little sequence similarity may suggest a third pasivirus species. Just as bat kunsagivirus, bat crohivirus was also suggested to infect mammals according to the NCA. Thirdly, sapeloviruses make up a genus that comprises three species: *Sapelovirus A, Sapelovirus B* and *Avian sapelovirus* [34]. The novel sapelovirus is closely related to sapelovirus A1. In recent years, a number of sapelo-like viruses have been detected in the feces of bats, other mammals and birds [35–40]. Therefore, it is likely that bats are among the natural hosts of these viruses especially as the novel sapeloviruses identified in this study was predicted to have a mammalian host by the NCA. Due to the high diversity of sapeloviruses, a revision of the respective taxonomy is currently in progress.

Bat iflavirus is closely related to slow bee paralysis virus, belonging to the family *Iflaviridae*. The latter virus infects the honey bee and together with Varroa destructor virus may be critical for colony mortality [41]. All currently known iflaviruses infect insects. In addition, NCA suggests that the bat iflavirus has an insect host, and was therefore diet derived; despite the high number of iflavirus reads identified in the sample (Table 1). Therefore we speculate that bat iflavirus was the result of the consumption of fruits or leaves containing insects, larvae or eggs infected with this virus, as has been suggested before [42].

The bat monocistronic posalivirus and fisalivirus gene arrangements correspond to that of the monocistronic marnavirus. The genome of posalivirus depicts a multidrug and toxic compound extrusion (MATE) domains usually found only in bacteria [31]. These proteins are involved in active efflux of drugs from the cell thereby enabling resistance [43]. Given that the e-value was only 3.95e^−03^, the presence of these domains requires confirmation. Fisalivirus does not have this domain and is more fisavirus-like. Posaviruses 1 and 2, fisavirus 1, husavirus, bat posalivirus, and fisalivirus do not only have a similar gene arrangement but also cluster together in the 3CD phylogenetic tree (Fig. 3, 4d). They have a wide vertebrate host range as they were detected in feces of pigs (posaviruses) [9, 10], humans (husaviruses) [11], the intestinal content of a carp (fisavirus) [8] and now in feces of bats. Apart from that, an RdRP-like sequence closely related to posavirus 1 was also found in a cDNA library generated from RNA of *Ascaris suum* [9], a nematode parasitizing the pig intestine. Due to its presence in this nematodes, Shan and colleagues [9] postulated that posaviruses may be infecting these nematodes in the intestine of pigs. Whether posavirus really infects the round worm may be questioned, but a contamination of *Ascaris* with a porcine virus appears to be an alternative explanation. Our NCA analysis classified bat posali-, fisali- and felisavirus as insect infecting picorna-like viruses. However, since the training dataset did not contain viruses infecting nematodes, no final conclusions on the host can be made. Despite the current uncertainties about the origin of these posa-like viruses, they may constitute a new family in the order *Picornavirales*.

Bat cripavirus is closely related to Himetobi P virus, an insect picorna-like virus isolated from the small brown planthopper, *Laodelphax striatellus* [44]*.* Furthermore, another cripavirus was recently described (NB-1/2011/HUN, KJ802403), possessing five rather than three VPg genes, in the guano from an insectivorous bat, *Pipistrellus pipistrellus* [45]. Genetic relatedness and NCA, seem to suggest that bat cripavirus might be an insect virus, and therefore diet was the most likely source.

Bat felisavirus has a dicistronic genome with gene organization similar to viruses of the *Dicistroviridae* but only part of one rhv conserved domain could be identified in a conserved domain search. Phylogenetic analyses of the pro-pol domains revealed clustering with the fesavirus 1 (detected in clinically healthy cats) as closest relative [7]. There is only a partial fesavirus genome available (KM017736) but this virus has apparently a monocistronic genome layout whereas bat felisavirus displays two ORFs (Fig. 1). It is unclear yet whether fesavirus of cats has a distinct genome organization or whether a previous sequencing artefact could explain this difference. The intergenic region of bat felisavirus is rather short (52–75 nt). Therefore, it is unlikely that a dicistrovirus-like IGR-IRES mediates translation initiation of ORF2. Although NCA indicates an insect host for the bat felisavirus, identification of more closely related viruses with known host species would be needed to further validate this finding.

Bat dicibavirus, and badiciviruses 1 and 2 are other virus with a dicistronic genome. In each case, the two ORFs are separated by an IGR of 196, 457 and 227 nt, respectively, and the ORF2 starts with a glycine codon, which is typical for IGR-IRES driven translation initiation. However, characteristic secondary structures were not yet identified. Phylogenetic analyses (Fig. 3, Additional file 1: Figure S1) show that bat dicibavirus clusters together with marine picorna-like viruses which all infect marine protists and diatoms, and therefore, together with labyrnavirus and bacillarnavirus may form a new family of viruses in the *Picornavirales.* Both bat badiciviruses cluster with two insect viruses, Aphis glycines virus and soybean-associated bicistronic virus. This implies that the origin of this virus in bat stool remains obscure and is probably dietary. NCA analysis groups them within the insect cluster thereby indicating that they may be indeed insect viruses originating from the diet. Further, these viruses may also constitute a novel family in the order *Picornavirales*.

## Conclusion

We were able to identify and characterize eleven novel and diverse RNA viruses of the order *Picornavirales.* This point to the considerable genetic variability of picorna-like viruses found in *Eidolon helvum* fruit bats. Bat kunsagivirus, crohivirus, and sapelovirus are clearly picornaviruses and this is consistent with the growing number of picornaviruses detected in bats. Many picornavirus infections cause a wide range of symptoms depending on virus-specific and host-specific factors. Whether the affected bats were diseased, is unclear. The captured bats of the present study were apparently healthy without any obvious clinical symptoms. This, however, does not exclude the potential of these picornaviruses to induce diseases in their natural hosts or in other species after spillover infections.

Given that many severely pathogenic zoonotic viruses have been traced to originate from bats, the knowledge obtained in this study is valuable to rapidly trace potential future zoonotic infections from bats to humans. Furthermore, all bat samples analyzed here were collected from regions where people hunt, sell and eat bats, so coupled with the fact that the *Picornaviridae* family is made up of a vast amount of highly divergent human pathogens, zoonoses therefore are not unlikely. However, the potential of these viruses to infect humans remains to be investigated.

Bat iflavirus and cripavirus are viruses of the family *Iflaviridae and Dicistroviridae*, respectively, and are likely of an insect dietary origin. Bat posalivirus, bat fisalivirus, bat felisavirus, bat dicibavirus and bat badiciviruses 1 and 2, are unassigned members of the order *Picornavirales*, and their natural hosts is currently unclear, and remains to be determined.

## Additional file

Additional file 1: Table S1. List of primers for genome completion. **Table S2.** Reference sequences used for composition analysis and differentiation of host groups. **Figure S1.** Phylogenetic relationships of orthologous capsid proteins: (A) P1 of picornaviruses (either 1A-1B-1C-1D or 1AB-1C-1D), (B) VP2-VP4-VP3-VP1 of bacillarnavirus, labyrnavirus and other viruses with a similar genome organization, (C) VP2-VP4-VP3-VP1 of dicistroviruses, (D) three capsid protein domains of yet unassigned, monocistronic viruses with C-terminal structural proteins (posa-, fisa-, husa-, bat posali- and fisalivirus, drosophila Pow Burn virus), and (E) VP2-VP4-VP3-VP1 domains of iflaviruses. Bat viruses of this study are indicated by filled triangles. Unassigned viruses are printed in blue. Bars indicate nucleotide substitutions per site. The trees were inferred with MrBayes 3.2. Numbers at nodes indicate posterior probabilities obtained after 4,250,000 generations (A), 3,000,000 generations (B), and 1,000,000 generations (C, D, E). The GTR + G + I substitution model was used for (A, B), the GTR + G model was used for (C, D, E). (DOCX 188 kb)

## Abbreviations

CDD: Conserved domains database
cDNA: Complementary DNA
CRPV domain: Cricket paralysis virus capsid domain
Hel-Pro-Pol domain: Helicase-protease-polymerase domain
HIV: Human immunodeficiency virus
IGR: Intergenic region
IRES: Internal ribosome entry site
LDA: Linear discriminant analysis
NCA: Nucleotide composition analysis
NetoVIR: Novel enrichment technique of VIRomes
ORF: Open reading frame
Rhv domain: Rhinovirus capsid domain
RNA: Ribonucleic acid
UTR: Untranslated region
