# Supplementary material for: Highly diverse population of Picornaviridae and other members of the Picornavirales, in Cameroonian fruit bats
Source: BMC Genomics. 2017 Mar 23;18:249. doi: 10.1186/s12864-017-3632-7 (PMC5364608; doi:10.1186/s12864-017-3632-7)

**Table 1:** List of primers for genome completion

| **Virus** | **Name of primer** | **Sequence (5’→3’)** |
| --- | --- | --- |
| Bat Kunsagivirus | Kunsagi30_2546-2568_F | GAT GTT GAA AGG AAC CCA GG |
|  | Kunsagi30_3633-3652_R | AGT AAC AAA GTG ACC ACG GG |
|  | Kunsagi30_5893-5914_F | GTC CCA AAA CAA AAT ACA TGC C |
|  | Kunsagi30_6296-6275_R | TCC ATC AAA TTT ACC TTG CTC C |
| Bat Fisalivirus | Fisa60_1625-1645_F | ACAGACAATGGCTACATTGCT |
|  | Fisa60_1709-1730_R | TCT TCA CAT TAA CGC AGA TCC T |
|  | Fisa60_3293-3314_F | TTG TTG CCA TAT ATG TGA CAC C |
|  | Fisa60_3563-3583_R | TCC AAT TCT TGA CAG TGT AGC |
|  | Fesa60_5440-5462_F | TTA TCT GAG GCA TTC TTT CAT GG |
|  | Fesa60_5758-5777_R | TTC ACT GAG GTT TGA CTG GG |
| Bat Dicibavirus | Diciba30_5749-5772_F | CTT GCT CAA ATT ACA AAT GTA ACG |
|  | Diciba30_6425-6405_R | TTG ATG TTA CCC AAT ACA TTA CCA |

**Table 2:** Reference sequences used for composition analysis and differentiation of host groups

A: Reference sequences used for composition analysis

*Mammals:*

AB205396, AB252582, AB426611, AB678778, AF039205, AF081485, AF083069, AF123432, AF123433, AF162711, AF311939, AF524867, AJ005695, AJ132961, AJ577589, AJ889918, AM235750, AY302539, AY302540, AY302541, AY302542, AY302543, AY302544, AY302545, AY302546, AY302547, AY302548, AY302549, AY302550, AY302551, AY302552, AY302553, AY302554, AY302555, AY302556, AY302557, AY302559, AY429470, AY508697, AY751783, AY843297, AY876912, AY876913, DQ358078, DQ473485, DQ473486, DQ473488, DQ473489, DQ473490, DQ473491, DQ473492, DQ473493, DQ473494, DQ473497, DQ473499, DQ473500, DQ473504, DQ473505, DQ473506, DQ473507, DQ473508, DQ473510, DQ473511, DQ995634, DQ995640, DQ995647, EF051629, EF107097, EF173414, EF173415, EF173420, EF173423, EF173425, EF552688, EF552689, EF552690, EF552691, EF552692, EF552693, EF552694, EF552695, EF552696, EF552697, EF555645, EU140838, EU716175, EU815052, FJ445111, FJ445112, FJ445113, FJ445114, FJ445116, FJ445118, FJ445119, FJ445120, FJ445121, FJ445122, FJ445123, FJ445124, FJ445125, FJ445126, FJ445127, FJ445128, FJ445129, FJ445130, FJ445131, FJ445132, FJ445133, FJ445134, FJ445135, FJ445136, FJ445138, FJ445140, FJ445141, FJ445142, FJ445143, FJ445144, FJ445145, FJ445146, FJ445147, FJ445148, FJ445149, FJ445150, FJ445151, FJ445152, FJ445153, FJ445154, FJ445155, FJ445156, FJ445157, FJ445160, FJ445161, FJ445162, FJ445163, FJ445164, FJ445165, FJ445167, FJ445168, FJ445169, FJ445170, FJ445171, FJ445172, FJ445173, FJ445174, FJ445175, FJ445176, FJ445178, FJ445179, FJ445180, FJ445181, FJ445182, FJ445183, FJ445185, FJ445186, FJ445187, FJ445188, FJ445189, FJ445190, FM955278, GQ249161, GQ323774, GQ865517, HM185056, HM777023, HQ400942, HQ702854, HQ728259, HQ728260, HQ728261, HQ728262, HQ875059, JF905564, JN088541, JN379039, JQ277724, JQ818253, JQ911763, JQ975417, JX050181, JX174177, JX262382, JX961709, JX982257, KF312882, KF422142, KF874626, KF958308, KF990476, KJ857508, KM609480, KP036483, KP345887, LK021688, NC_001366, NC_001430, NC_001472, NC_001489, NC_001490, NC_001612, NC_001859, NC_001897, NC_001918, NC_001617, NC_002058, NC_003976, NC_003985, NC_003987, NC_003988, NC_004421, NC_004441, NC_004451, NC_008714, NC_009891, NC_009996, NC_010354, NC_010810, NC_011349, NC_011829, NC_012798, NC_012800, NC_012801, NC_012802, NC_012957, NC_012986, NC_015936, NC_016156, NC_016769, NC_016964, NC_018226, NC_018668, NC_021178, NC_021220, NC_021482, NC_022802, NC_023422, NC_023637, NC_023638, NC_023984, NC_024070, NC_024073, NC_025114, NC_025474, NC_025675, NC_025961, NC_026249, NC_026314, NC_026315, NC_026316, NC_027054, NC_027818, NC_027918, NC_027919, NC_028240, NC_028363, NC_028364, NC_028365, NC_028479, NC_028981, NC_029854, NC_029905, V01149, X00925, X56019, X67706, X77708, X84981, X92886, NC_026470, NC_030843, NC_028366.

Insects:

AB766259, HM237361, JF720348, NC_001834, NC_001874, NC_002066, NC_002548, NC_003113, NC_003779, NC_003781, NC_003782, NC_003783, NC_003784, NC_003924, NC_004365, NC_004807, NC_004830, NC_005092, NC_006559, NC_008029, NC_009025, NC_009530, NC_014137, NC_016405, NC_021566, NC_021567, NC_022611, NC_023021, NC_023022, NC_023483, NC_023627, NC_023676, NC_024016, NC_024497, NC_025788, NC_025835, NC_026250, NC_026733, NC_027713, NC_027917, NC_030115.

Plants:

NC_001632, NC_003003, NC_003445, NC_003495, NC_003544, NC_003549, NC_003615, NC_003621, NC_003626, NC_003628, NC_003693, NC_003738, NC_003785, NC_003787, NC_003791, NC_003799, NC_003839, NC_005266, NC_005289, NC_006056, NC_006271, NC_006964, NC_008182, NC_009013, NC_010709, NC_010987, NC_011189, NC_013218, NC_015414, NC_015492, NC_016443, NC_020897, NC_022004, NC_023016, NC_022798, NC_027915, NC_027926, NC_028139, NC_029036, NC_025479.

B: Differentiation of host groups using mononucleotide and dinucleotide frequency

Mammal Unassigned Correct

Mammal 270 16 94%

Insect 41 3 93%

Plant 40 2 95%

**Figure 1:** Phylogenetic relationships of orthologous capsid proteins: (A) P1 of picornaviruses (either 1A-1B-1C-1D or 1AB-1C-1D), (B) VP2-VP4-VP3-VP1 of bacillarnavirus, labyrnavirus and other viruses with a similar genome organization, (C) VP2-VP4-VP3-VP1 of dicistroviruses, (D) three capsid protein domains of yet unassigned, monocistronic viruses with C-terminal structural proteins (posa-, fisa-, husa-, bat posali- and fisalivirus, drosophila Pow Burn virus), and (E) VP2-VP4-VP3-VP1 domains of iflaviruses. Bat viruses of this study are indicated by filled triangles. Unassigned viruses are printed in blue. Bars indicate nucleotide substitutions per site. The trees were inferred with MrBayes 3.2. Numbers at nodes indicate posterior probabilities obtained after 4,250,000 generations (A), 3,000,000 generations (B), and 1,000,000 generations (C, D, E). The GTR+G+I substitution model was used for (A, B), the GTR+G model was used for (C, D, E).


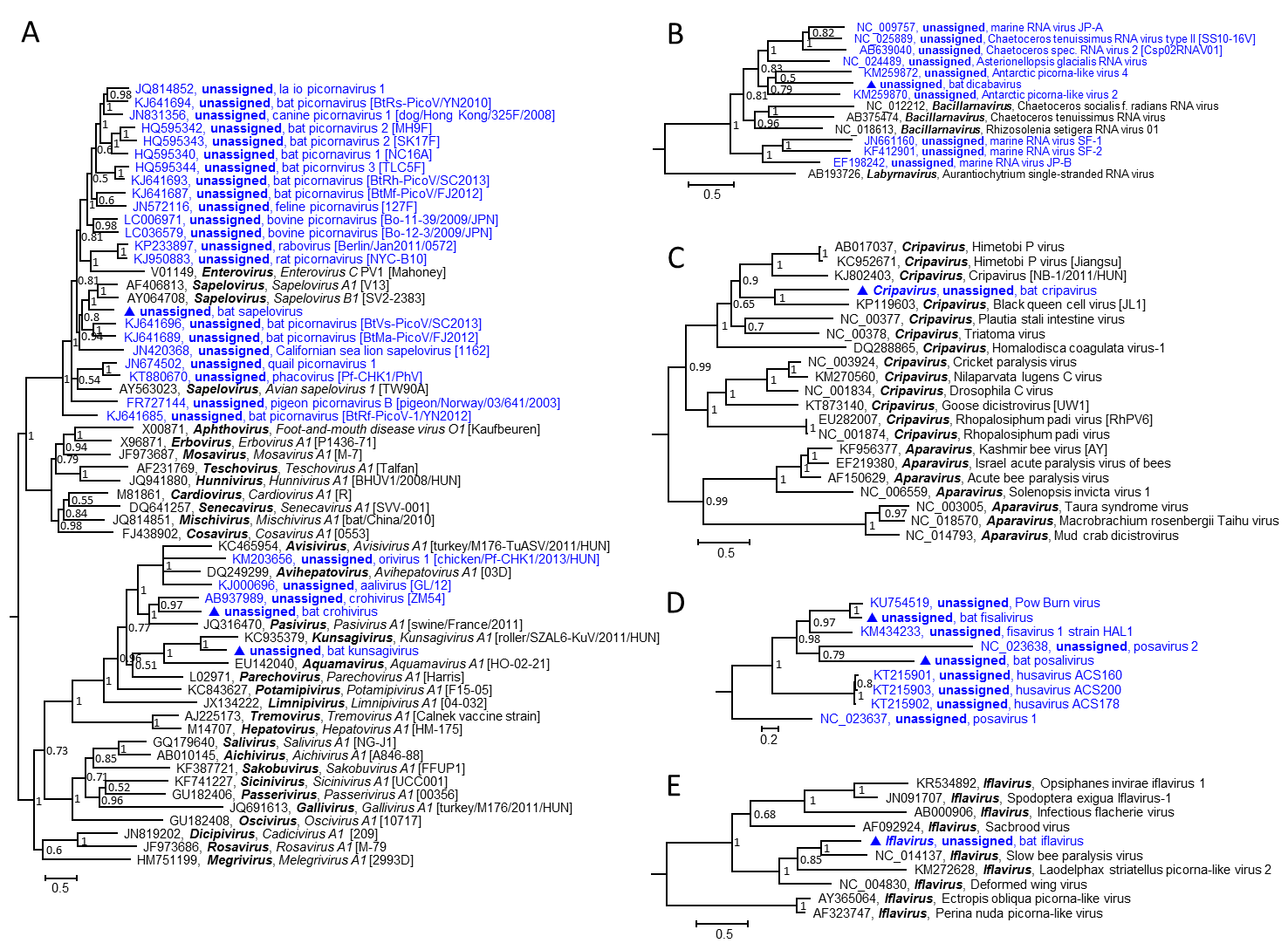

Supplement: Additional file 1: Table S1. — List of primers for genome completion. Table S2. Reference sequences used for composition analysis and differentiation of host groups. Figure S1. Phylogenetic relationships of orthologous capsid proteins: (A) P1 of picornaviruses (either 1A-1B-1C-1D or 1AB-1C-1D), (B) VP2-VP4-VP3-VP1 of bacillarnavirus, labyrnavirus and other viruses with a similar genome organization, (C) VP2-VP4-VP3-VP1 of dicistroviruses, (D) three capsid protein domains of yet unassigned, monocistronic viruses with C-terminal structural proteins (posa-, fisa-, husa-, bat posali- and fisalivirus, drosophila Pow Burn virus), and (E) VP2-VP4-VP3-VP1 domains of iflaviruses. Bat viruses of this study are indicated by filled triangles. Unassigned viruses are printed in blue. Bars indicate nucleotide substitutions per site. The trees were inferred with MrBayes 3.2. Numbers at nodes indicate posterior probabilities obtained after 4,250,000 generations (A), 3,000,000 generations (B), and 1,000,000 generations (C, D, E). The GTR + G + I substitution model was used for (A, B), the GTR + G model was used for (C, D, E). (DOCX 188 kb) [file 12864_2017_3632_MOESM1_ESM.docx]
